# Book Review

**Published:** 1911-06

**Authors:** 


**A Prescriber's Companion.** By THOMAS D. SAVILL, M.D. Fourth Edition. Revised by the AUTHOR, assisted by CHARLES F. HARFORD, M.D. Pp. 59. London: Henry J. Glaisher. 1910.—This booklet, revised by the request of and approved by the late Dr. Savill, is a *multum in parvo,* which should be very useful to those who in these days place their trust for the most part in vaccines, and neglect the art of prescribing drugs as they have been used in days which are passing. The prescriptions are founded on the accumulated experience of a lifetime, and much miscellaneous information in Part II, on disinfection, antidotes, infant feeding, contagious diseases, etc., and vaccines, may help a tyro out of many difficulties.

**Alcohol and Alpinism.** By Dr. L. SCHNYDER. Authorised translation by S. G. RICHARDS. Pp. 83. Edinburgh: William Green and Sons. 1910.—In a small volume we get the results of the author's investigations into the value of alcohol to climbers. Having his own opinions as the result of laboratory experiments, he obtained the opinions of over 500 prominent Alpinists of all nationalities, most of them scientific or medical men. The book is chiefly composed of these opinions and the author's commentary. Though at first sight such a book may seem unnecessary, such is by no means the case. Climbing is an increasingly popular sport. And in no other sport are the details of equipment so vitally important. So serious, if not avoided, are some of the dangers of the finest of all sports that it is only strict attention to every factor which makes for safety that ensures it being a sport for prudent men. The verdict of the majority is distinctly against the use of any form of alcohol while actually climbing. The stimulus is very short lived, and the subsequent depression even greater than at ordinary levels. Cold and exhaustion are best met by suitable feeding. Death from exposure on the mountain-side, as the result of a storm or being benighted, would be far less common if climbers carried extra Shetland wool underclothing, and left their brandy flasks below. Nothing is more certain than that most Alpine accidents are preventable, and mostly the results of carelessness. Alcohol undoubtedly lessens the caution which should form a large part of the mountaineer's disposition, and its use is to be discouraged. The traditional bottle of champagne on the summit is by no means without its dangers. Too many accidents have occurred shortly after the descent has commenced. We are the last to deny that alcohol has some uses. Under certain circumstances a temporary stimulant is most useful. A young novice this summer, as the result of his own carelessness, fell into a crevasse on the Pic D'Arolla. He was extricated with difficulty after hanging on the rope for fifteen minutes. With fright and cold his condition was pitiable. He talked of immediate descent. But as this would have been dangerous, and the remainder of the climb was easy, he was well stimulated, and recovered enough pluck to reach the summit and descend by a safer way. Also after a trying day the use of a little wine to aid digestion is often quite wise as food is the best antidote to fatigue. This volume is a useful addition to the Alpine Library.

**Johns Hopkins Hospital Reports.** Vol. XV. Baltimore: The Johns Hopkins Press. 1910.—This volume testifies to the excellence of the work done at the Johns Hopkins Hospital, and provokes the thought that what is being done at Baltimore might be done in Bristol. The requisite clinical material is to hand, but the records kept are not yet good enough, and not everyone in a position to make use of case records realises as yet that it is his duty to do so *pro bono publico.* The first paper, by J. H. Mason Knox and Edwin H. Schorer, attempts to correlate clinical and bacteriological dates in the summer diarrhœa of infants. Some variety of B. dysenteriæ were present in 58 per cent. of the seventy-four cases studied; in 22.9 per cent. streptococci were found. Treatment with serum was not satisfactory, probably because the usual serum corresponds with the Shiga variety of B. dysenteriæ, which was not found many times. The worst cases were those in which more than one type of dysentery bacillus was recovered from the stools; those with streptococci only were not severely ill. The mortality rate was more than twice as high in infants who had been fed on condensed milk as in those fed on cows' milk. Twelve papers next following deal with various aspects of lobar pneumonia and pneumococcal infection. These yield a vast mass of statistical and other data, of which a few may be quoted. The mortality rate in Baltimore is high, 25 per cent. if terminal pneumonias be included; in eighty-seven cases subjected to open-air treatment the mortality was 10 per cent. In the *post-mortem* cases the right lung was pneumonic four times as often as the left. There is practically always some leucocytosis; its absence is usually a bad sign, liable to be noted in cases of terminal pneumonia. An article on the successful termination of pneumonia, by C. P. Emerson, emphasises the risk of severe and even fatal collapse in crisis. The pneumococcal type of endocarditis is, as H. T. Marshall shows, particularly prone to attack the aortic and tricuspid rather than the mitral valves. J. A. Chatard's paper on acute pericarditis complicating lobar pneumonia shows that it is a serious feature, and that its origin lies at least as often in blood infection as in direct transmission from pleura to pericardium. In Thomas McCrae's paper on empyema complicating pneumonia it is somewhat astonishing to read that the heart was not displaced to any extent in a single case. Steiner reports three cases of venous thrombosis in 658 cases of pneumonia, all in the veins of the lower limb, and summarises the literature. The subject of pneumococcal arthritis is dealt with by C. P. Howard, and F. M. Hanes describes twenty-five cases of pneumococcal meningitis, all fatal. The clinical picture varies, but presents no special feature apart from the insidious onset. Delayed resolution in the pneumonic lung does not, according to Thomas McCrae, occur specially in feeble subjects; the cautious use of X.-rays is of distinct benefit. The other two papers are very long. The first is a review of 550 cases of skin transplantation, by John Staige Davis, with a full account of the technique in use at the Johns Hopkins Hospital, and a good summary of the literature. F. J. Sladen, in the last paper, gives a very complete description of experiences in the treatment of meningococcal meningitis. Of twenty-three cases treated with Flexner's serum only fourteen died; while of twenty-three cases treated before the introduction of serum twenty-one were fatal. This paper should be consulted by anyone who has to treat cases of cerebrospinal meningitis.

**Some Considerations of Medical Education.** By S. Squire Sprigge, M.A., M.D. Pp. x., 99. London: Baillière, Tindall & Cox. 1910.—We have in this small work a most interesting and lucid account of the many changes which have taken place in the education of the medical student in recent years. The ever increasing amount of special knowledge which the rapid advances of science have made it necessary for the embryo medico to acquire has rendered some rearrangement of the curriculum imperative. The author explains that the lightening of the burden is being obtained by, on the one hand, an earlier specialisation of education, so that the elementary science subjects are being taught at the Public Schools, and on the other by a lengthening of the curriculum. The arguments in favour of these alternatives are given in some detail. Due notice is taken of the relative disadvantages under which the London student suffers in obtaining a medical degree. The question of the advisability of a one portal system is also discussed.

The author insists that the General Medical Council, although labouring under difficulties, not the least of which is the fact that the registration of students is not compulsory, has done and is doing good work. We can heartily recommend this book to the large class to whom the subject is of interest as an unbiased and practical view of the various problems involved.

**Meningitis, Sinus Thrombosis, and Abscess of the Brain.** By JOHN WYLLIE, M.D. Pp. ix., 258. London: H. K. Lewis. 1911.—The difficulties in the early differential diagnosis of intracranial infections fully justify the appearance of a work of this nature, but we do not think that the author has been altogether successful in the task which he has set himself. The general symptomatology is clearly given and is that generally accepted, but when special points are treated the same success is not evident. The anatomy of the petrous bone has always been a stumbling-block, but such a statement as "injury to the auditory nerve in the Fallopian tube" is topographically misleading. The use of the word tympanum where tympanic membrane is meant is also confusing. The description of Bárány's (here spelt Bardny) vestibular test is insufficient in detail to make it useful to those who have never seen it employed. Spinal puncture is well given, but the inclusion of spinal anæsthesia in a work of this nature is a step of doubtful utility. The appendix on the nasal accessory sinuses, in which œdema and swelling of the cheek are given as prominent signs in antral suppuration, and the sphenoidal sinus is made to open into the superior meatus, is scarcely sufficiently full or accurate to serve a useful purpose.

**Clinical Pathology in Practice.** By THOMAS T. HORDER, M.D., F.R.C.P. Pp. viii., 216. London: Oxford Medical Publications. 1910.—The aim of this book is to lay before the practitioner the various modern methods which may be used in the clinical elucidation of disease from a pathological aspect, while not being in any sense a systematic account of pathology. Good descriptions are given of how the necessary materials are to be collected, and the interpretations to be derived from investigating these are fully considered. Chapters are devoted to the examination of the different fluids which may be obtained by puncture, while others are devoted to such subjects as the diagnosis of tuberculosis and its specific treatment, to the various specific serum tests, to vaccine therapy, to the investigation of fever which is not associated with other physical signs. Useful illustrations are inserted throughout the text. The book is one which should be of much value to all who are not fully conversant with such procedures.

